# Facile Synthesis of a Novel AgIO_3_/CTF Heterojunction and Its Adsorption–Photocatalytic Performance with Organic Pollutants

**DOI:** 10.3390/toxics12020133

**Published:** 2024-02-06

**Authors:** Liqiang Shen, Tingting Ye, Yehui Chen, Bei Chu, Hui Chen, Jinxing Hu, Yan Yu

**Affiliations:** 1 Ningbo Key Laboratory of Agricultural Germplasm Resources Mining and Environmental Regulation, College of Science and Technology, Ningbo University, Cixi 315300, China; slq@shuiyigroup.com (L.S.); ytt_0105@126.com (T.Y.); 17857058735@163.com (Y.C.); chubei@nbu.edu.cn (B.C.); chenhui07@126.com (H.C.); hujinxing@nbu.edu.cn (J.H.); 2 Shui Yi Environmental Protection Group Co., Ltd., Ningbo University, Cixi 315300, China

**Keywords:** AgIO_3_/CTF, heterojunction, photocatalysis, degradation, organic pollutants

## Abstract

With the development of modern industry, the issue of water pollution has garnered increasing attention. Photocatalysis, as a novel green environmental technology that is resource-efficient, environmentally friendly, and highly promising, has found extensive applications in the field of organic pollutant treatment. However, common semiconductor materials exhibit either a relatively low photocatalytic efficiency in the visible light range or an inefficient separation of photogenerated charges, resulting in their limited ability to harness solar energy effectively. Consequently, the development of new photocatalysts has become a pivotal focus in current photocatalysis research to enhance solar energy utilization. This research provides a brief explanation of the photocatalytic mechanism of the AgIO_3_/CTF heterojunction photocatalyst. Due to the localized surface plasmon resonance (LSPR) effect, the Ag nanoparticles demonstrate significant absorption in the visible light region, playing a crucial role in the highly efficient photocatalytic reduction of organic pollutants.

## 1. Introduction

In recent years, the proliferation of organic pollutants in aquatic environments has been on the rise, progressively accumulating along the food chain and culminating in adverse health effects upon ingestion [1]. The imperative challenge lies in the effective removal of these organic pollutants from water bodies. Nevertheless, conventional physical and chemical methodologies prove challenging in comprehensively eliminating or degrading the organic pollutants in water. Photocatalysis technology, as an advanced oxidation technique, has evolved into an independent and challenging field through the development of heterogeneous semiconductor photocatalysts capable of degrading organic pollutants under solar irradiation [2]. Semiconductor materials, extensively explored in the realm of photocatalysis, exhibit notable advantages, including efficient photoelectric conversion and commendable stability [3]. Beyond meeting the demands for the removal of organic pollutants, the quest for efficient and energy-conserving pollution degradation methods, coupled with the construction of adsorption–photocatalysis composite material systems, holds promising applications.

Silver iodate (AgIO_3_), a recently acclaimed and efficient photocatalytic material, boasts p-type semiconductor characteristics with a relatively narrow bandgap, determining its robust absorption in the visible light spectrum [4]. However, AgIO_3_ suffers from severe photocorrosion, compromising the catalyst’s stability and restraining its widespread adoption in photocatalysis technology. Research reveals that the formation of composite catalysts with other semiconductors effectively suppresses the photocorrosion of the AgIO_3_, elevating the catalyst’s stability and catalytic activity [5]. Additionally, surface plasmon resonance, as a promising photocatalytic technique, has garnered increasing attention in recent years. Covalent triazine frameworks (CTFs) are a type of covalent organic framework (COF), and they have attracted substantial research attention in recent years. A CTF, characterized by typical n-type semiconductor properties, forms a p–n heterojunction composite catalyst when combined with AgIO_3_, significantly amplifying the photocatalytic performance. The p–n heterojunction, endowed with a potent internal electric field, enhances the charge separation and transfer capabilities [6]. Materials with varying bandgap widths broaden the photoresponse range, thereby augmenting the photocatalytic efficiency. This study endeavors to construct AgIO_3_/CTF heterojunction catalysts through optimized synthesis methods to realize the synergistic adsorption–photocatalytic removal of trace antibiotics from potable water. This research first studied the structural characteristics of the composite system based on a survey of the literature with a basis in original research to determine the physical and chemical properties of the micro-interface of the material. Then, we selected typical antibiotics, studied the adsorption–photocatalytic performance and influencing factors of the composite materials, and used this feedback to optimize the adsorption–photocatalytic experimental conditions. Then, we studied the stability and reusability of the composite materials. Finally, experiments were conducted to verify the structure–activity relationship and the mechanism of the antibiotic removal through the AgIO_3_/CTF heterojunction adsorption–photocatalytic composite system.

## 2. Experimental Section

### 2.1. Materials

The chemical reagents that were used in the experiment in this paper were 98% 1,4-dicyanobenzene (DCB), which is produced by the Beijing Bailingwei Technology Co., Ltd. (Beijing, China); 99.0% bismuth nitrate pentahydrate and 99% carbamazepine, which are produced by the Shanghai McLean Biochemical Technology Co., Ltd. (Shanghai, China); and 98% trifluoromethanesulfonic acid (TFMS), 99.5% N, *N*-dimethylformamide (DMF), ethanol, nitric acid, ≥47% potassium bisulfate (PMS), 99% ammonium metavanadate, 96% sodium hydroxide, 98% norfloxacin, 99.0% acetaminophen, KIO_3_, and AgNO_3_, which are produced by the Shanghai Aladdin Biochemical Technology Co., Ltd (Shanghai, China). Ultrapure water was obtained from Milli-Q^®^ Ultrapure Water Purification Systems (Milford, MA, USA).

### 2.2. Preparation of the Photocatalysts

Preparation of AgIO_3_: the AgIO_3_ powder was prepared through the solid-state ion exchange method. KIO_3_ (1.51 g) and AgNO_3_ were mixed thoroughly in a mortar until uniform solid particles formed. The resulting white precipitate was washed with deionized water to dissolve any unreacted starting material.

Preparation of AgIO_3_/CTF: in total, 0.302 g of AgNO_3_ and 0.24 g of KIO_3_ were weighed and ground together with a CTF in a mortar. After grinding until completely mixed, the powder was transferred in the mortar to a 250 mL beaker, deionized water was added, and it was stirred for 5 min. Then, it was centrifuged in an ultra-high-speed refrigerated centrifuge with the rotation speed set to 12,000 r/min; it was then centrifuged and washed several times, and dried in a 60 °C oven, so that the x-AgIO_3_/CTF could be collected for later use.

This experiment used a xenon lamp as a light source to simulate sunlight with a 400 nm cut filter. First, the low-temperature thermostatic ring water pump was set to 25 °C, the solution (100 mL, 5 mg L^−1^) was measured and poured into a 200 mL quartz glass vessel, 40 mg of PMS was added, and it was stirred magnetically for 30 min. Then, 1 mL of the solution was extracted, filtered, and injected into the liquid phase vial.

### 2.3. Characterization of Photocatalysts

An X-ray powder diffraction (XRD) analysis of the catalysts was carried out using an X-ray diffractometer (XPert Pro MPD; PANalytical B.V., Malvern, UK) using Cu-Kα radiation generated at 45 kV and 40 mA. The morphologies of the catalysts were observed using field emission scanning electron microscopy (FESEM, S-4800; Hitachi, Japan) operated at an accelerating voltage of 4 kV. The transmission electron microscopy (TEM) was performed through Tecnai G2F30 S-Twin TEM (FEI, Amsterdam, The Netherlands). The X-ray photoelectron spectroscopy (XPS) experiments were performed on a Thermo Scientific ESCA-Lab-200i-XL spectrometer (Waltham, MA, USA) with monochromatic Al Alα radiation (1486.6 eV), and the C 1s and N 1s peak spectra were analyzed using XPS Peak 4.1 software. The Raman spectra were obtained using a Lab Ram HRUV Raman spectrometer (JDbin-yvon, Paris, France), and the laser excitation was provided by an Ar^+^ laser at a wavelength of 532 nm. The Fourier transform infrared spectroscopy (FTIR) spectra were recorded in the 4000–400 cm^−1^ region with a resolution of 4 cm^−1^ using a Nicolet Thermo NEXUS 670. The steady-state PL spectra were measured using a Hitachi F-4600 fluorescence spectrophotometer (Hitachi, Japan). The data were recorded and analyzed by means of built-in software supplied by Edinburgh Instruments.

### 2.4. Adsorption–Photocatalytic Degradation of Antibiotics

In this experiment, a xenon lamp was used as a light source to simulate sunlight. The reaction solution (100 mL, 5 mg L^−1^) was measured into the reactor and 40 mg PMS was added with magnetic stirring and a constant temperature of 25 °C. In total, 50 mg of photocatalyst was weighed and stirred in a dark state for 30 min, then the xenon lamp was turned on and 1 mL of liquid sample was taken successively under the preset time intervals and injected into the liquid phase vial after filtration. Four antibiotics were degraded with each photocatalyst (paracetamol solution 100 mL, 5 mg L^−1^; carbamazepine solution 100 mL, 5 mg L^−1^; phenol solution 100 mL, 5 mg L^−1^; and p-chlorophenol solution 100 mL, 5 mg L^−1^).

### 2.5. Radical Quenching Experiment

Through the addition of different free radical scavengers, disodium EDTA-2Na, methanol (MeOH), p-benzoquinone (PBQ), and histidine (his), combined with electron paramagnetic resonance (ESR) analysis, the main active species and photocatalytic degradation mechanism in the catalytic reaction system were determined. The aim was to explore the action mechanism of the AgIO_3_/CTF heterojunction photocatalyst in the system, in order to provide a theoretical basis for the construction of an efficient adsorption–photocatalysis dual function composite system that removes the organic pollutants in water.

## 3. Results and Analysis

**Figure 1:**
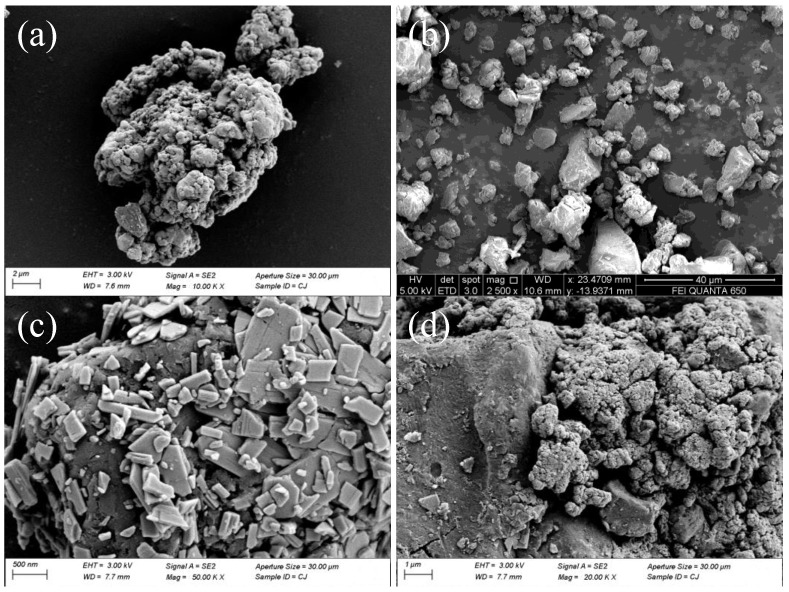
SEM images of (**a**) AgIO_3_, (**b**) CTF-N, and (**c**) AgIO_3_ and (**d**) 0.25AgIO_3_/CTF.

Morphology has an important impact on the specific surface area and surface defects of the material and has an impact on the photocatalytic performance or adsorption performance of the material. Therefore, the photocatalytic performance of the material can be better studied by observing the morphology of the sample. Figure 1a,b are SEM images of CTF-B and CTF-N. It can be seen from the figure that the CTF-B is lumpy and irregular in shape, while the CTF-N is an accumulation of particles smaller than the CTF-B and has a rough surface. Figure 1c,d show the SEM images of the AgIO_3_ and AgIO_3_/CTF. Among them, the AgIO_3_ is in the form of flakes with an uneven shape, a thickness of about 20–60 nm, and a maximum lateral width between 200–800 nm. In addition, it can be seen that AgIO_3_ nanosheets are distributed on the surface of the CTF-N.

**Figure 2:**
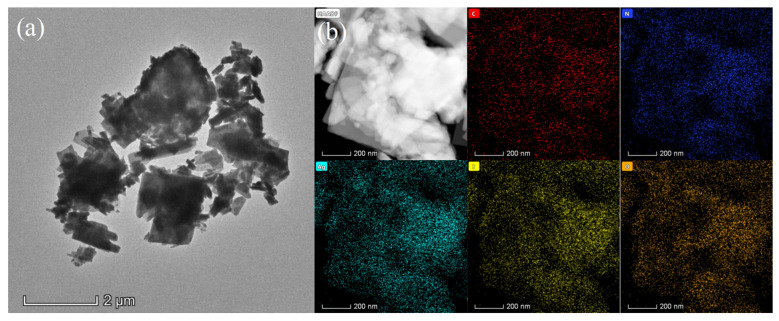
TEM image of (**a**) 0.25AgIO_3_/CTF; and (**b**) a typical high-angle annular dark-field (HAADF) image of 0.25AgIO_3_/CTF and the corresponding EDS mapping images of C, N, Ag, I, and O.

**Figure 3:**
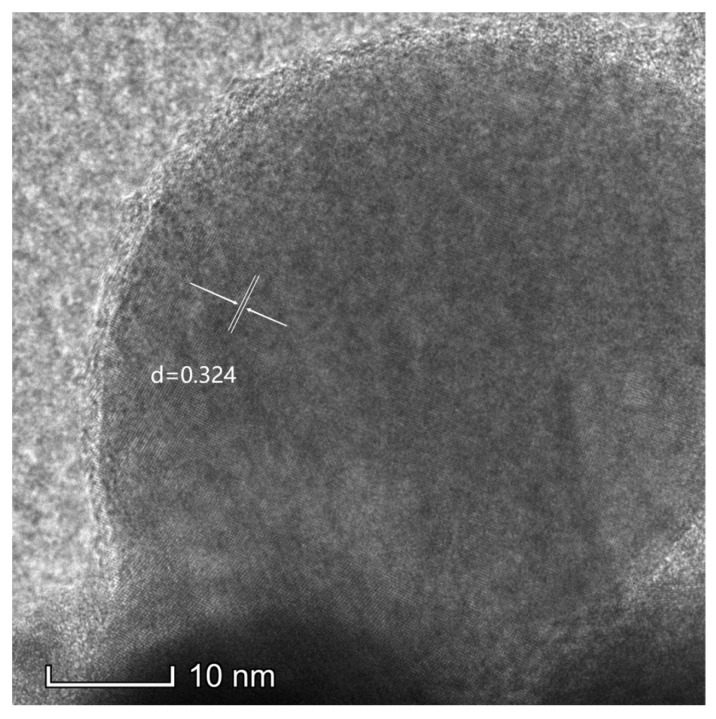
The HRTEM image of 0.25AgIO_3_/CTF.

A TEM is a more intuitive way to observe the internal structure and spatial distribution of the samples. Figure 2 is the TEM image and elemental scanning results of 0.25AgIO_3_/CTF. It can be intuitively seen that AgIO_3_ nanosheets are distributed on the surface of an approximately single-layer and highly dispersed CTF-N with a maximum lateral width between 200–800 nm, which is consistent with the sample appearance shown in the SEM image. In Figure 2, the CTF-N has a translucent structure, indicating that its thickness is very thin. The flaky AgIO_3_ and the flaky CTF-N were organically combined to successfully construct the AgIO_3_/CTF composite photocatalyst. It can be clearly seen that the constituent elements of the AgIO_3_, Ag, I, and O, are evenly distributed on the constituent elements, C, O, and N, of the CTF-N. However, because the AgIO_3_ is highly sensitive to the damage caused by electron transmissions to internal atoms, we cannot further analyze the microstructure of the composite photocatalyst using a polymer transmission electron microscope. The HRTEM image (Figure 3) of the 0.25AgIO_3_/CTF pictures lattice fringes with a spacing of 0.324, which correspond well to the {220} facet of the AgIO_3_.

**Figure 4:**
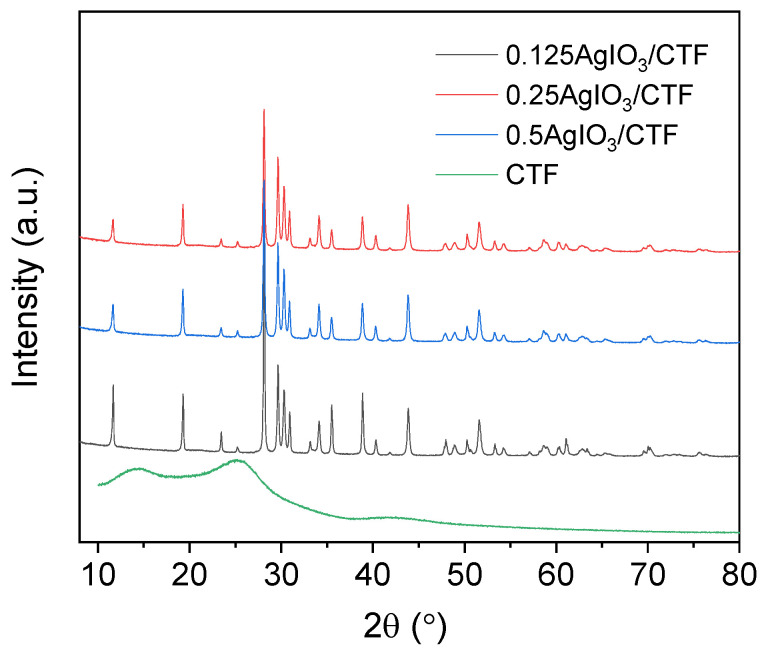
XRD patterns of x-AgIO_3_/CTF.

An XRD can effectively analyze the phase composition and phase purity of the samples. XRD tests were conducted on the x-AgIO_3_/CTF. Figure 4 is the XRD pattern of the 0.125AgIO_3_/CTF, 0.25AgIO_3_/CTF, and 0.5AgIO_3_/CTF. It is observed from Figure 3 that as the AgIO_3_ content in the sample increases, the diffraction peak of the AgIO_3_ in the sample gradually becomes apparent. The diffraction peak of the AgIO_3_ in the 0.125AgIO_3_/CTF sample is the most obvious, and the diffraction peak of the AgIO_3_ in the 0.25AgIO_3_/CTF and 0.5AgIO_3_/CTF can also be clearly observed. Therefore, it is believed that AgIO_3_ adheres to the CTF to form an AgIO_3_/CTF composite compound, which further proves the successful synthesis of 0.125AgIO_3_/CTF, 0.25AgIO_3_/CTF, and 0.5AgIO_3_/CTF.

**Figure 5:**
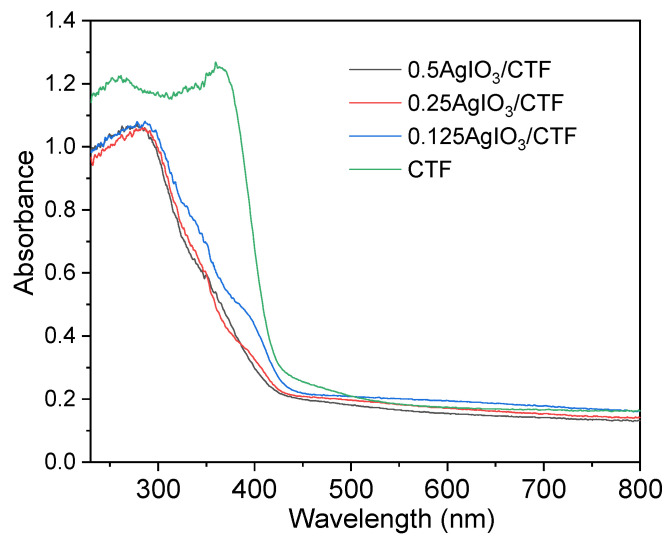
UV–vis absorption spectra of CTF and x-AgIO_3_/CTF.

In order to analyze the light absorption capacity and optical band gap of the x-AgIO_3_/CTF composites, UV-vis tests were conducted. Figure 5 shows the absorption in the visible light region, which demonstrates that the UV-vis diffuse reflection spectrum of the x-AgIO_3_/CTF composite material is in the wavelength range of 230–800 nm. It can be seen from the figure that the x-AgIO_3_/CTF absorbs in the range of 230–430 nm and has an obvious AgIO_3_ characteristic absorption peak in the 285 ± 5 nm region. The light absorption intensity of this characteristic absorption peak is 1.08. The absorbance of x-AgIO_3_/CTF in the 230–800 nm range is higher than that of pure AgIO_3_. This is due to the interaction generated by the formation of a heterostructure between the AgIO_3_ and CTF, which effectively enhances the separation of electron–hole pairs and promotes the band gap transition of photogenerated electrons, thus enhancing the absorption in the visible light region [7]. Theoretically, as the intensity of the light absorption by a catalyst increases, the absorbed light energy increases, which increases the rate of generating excited electrons and holes, thereby increasing the photocatalytic efficiency [8]. The absorption band edge of the x-AgIO_3_/CTF is around 430 nm, which is significantly “red-shifted” compared with a sample AgIO_3_ and shows a good optical response capability. This shows that the composite system is conducive to improving the material’s utilization of sunlight.

**Figure 6:**
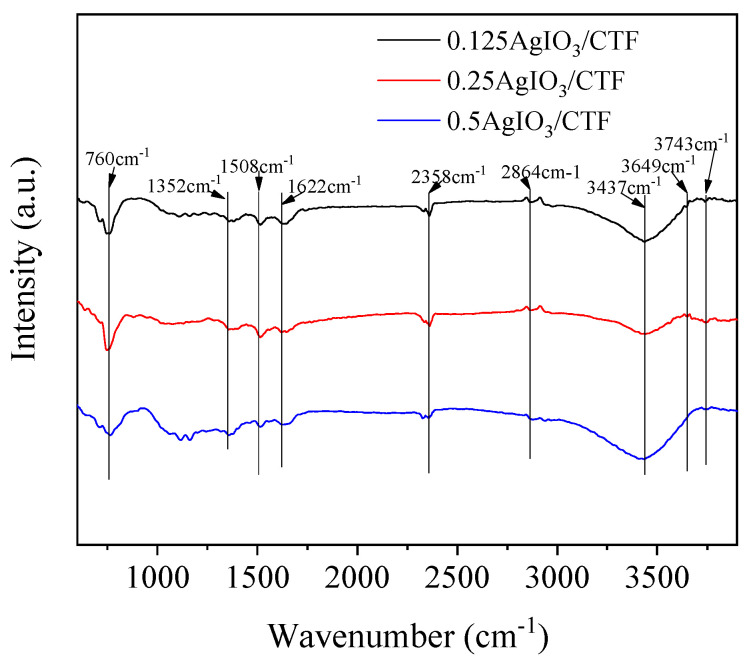
FTIR spectra of x-AgIO_3_/CTF.

The molecular structure and functional groups of the synthetic materials were analyzed via a FTIR spectroscopy. Figure 6 shows the FTIR of the x-AgIO_3_/CTF. Several absorption peaks can be seen from the figure, among which the peak at 760 cm^−1^ corresponds to the stretching vibration of the I=O bond [9]. The absorption peak located at 1350–1650 cm^−1^ corresponds to the characteristic peak caused by the vibrational stretching of the aromatic C=N bond formed by the triazine ring. The peak located near 2864 cm^−1^ corresponds to the symmetric stretching vibration peak of the CH_3_, and the peaks near 3437 cm^−1^, 3649 cm^−1^, and 3743 cm^−1^ are considered to be the characteristic stretching vibration peaks of hydroxyl groups on the sample surface [10].

**Figure 7:**
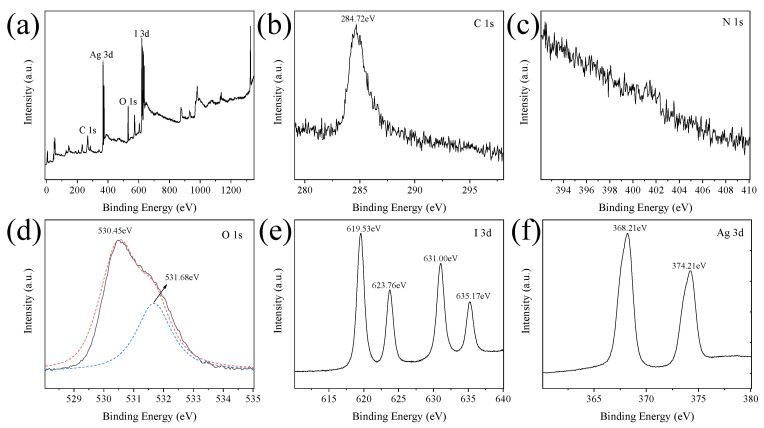
XPS spectra of (**a**) 0.25AgIO_3_/CTF, (**b**) C 1s, (**c**) N 1s, (**d**) O 1s, (**e**) I 3d, and (**f**) Ag 3d.

We can compare this with the XPS of 0.25AgIO_3_/CTF (Figure 7a). Obviously, the peaks of C 1s, N 1s, O 1s, I 3d, and Ag 3d can be found in their measured spectra. The C element (Figure 7b) and N 1s (Figure 7c) originates from the base of the XPS instrument, and no other peaks were observed, indicating that the prepared sample is of a high purity. The deconvolution of the O 1s peak, with two characteristic absorption peaks at 530.45 and 531.68 eV (Figure 7d), was attributed to the lattice oxygen atom (I-O) and the surface-adsorbed oxygen. The peaks at 619.53 eV, 623.76, 631.00 and 635.17 eV (Figure 7e) can be attributed to the I 3d5/2 orbital and I 3d3/2 orbital, respectively, which are the characteristic peaks of I^5+^ in AgIO_3_. The peaks at 368.21 eV and 374.21 eV (Figure 7f) can be assigned to the Ag 3d5/2 orbital and the Ag 3d3/2 orbital, respectively. These results further prove that the chemical composition of 0.25AgIO_3_/CTF remains unchanged after being used for degradation. The 0.25AgIO_3_/CTF sample contains Ag, I, N, C, and O elements, and the valence state of each element is consistent with the valence state of each element in the 0.25AgIO_3_/CTF. This once again proved the successful preparation of the 0.25AgIO_3_/CTF sample [11].

**Figure 8:**
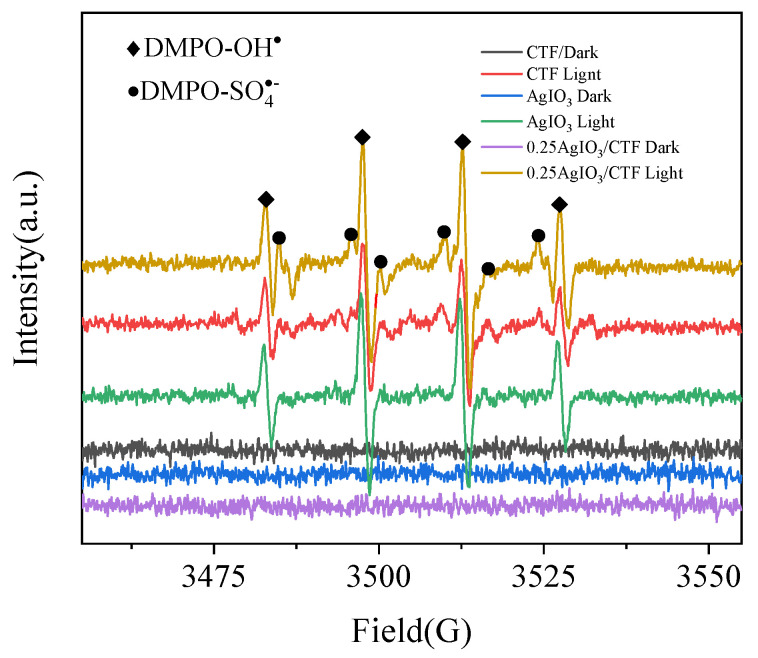
EPR spectra of AgIO_3_ and 0.25AgIO_3_/CTF using DMPO as the spin-trapping agent in water.

Figure 8 shows the EPR spectra of the CTF, AgIO_3_, and 0.25AgIO_3_/CTF samples under light conditions and dark conditions. It can be seen that there are four strong symmetry signals, and the existence of the g value proves that there are vacancies formed by unpaired electrons on the aromatic ring. Under dark conditions, compared with the AgIO_3_, the EPR curve intensity of the 0.25AgIO_3_/CTF is stronger. This shows that there are more unpaired electrons in the 0.25AgIO_3_/CTF sample, and it also indicates that the sample has a defect structure, which is conducive to the migration of photogenerated carriers. When the CTF, AgIO_3_, and 0.25AgIO_3_/CTF were irradiated with visible light, the EPR signal intensity of the 0.25AgIO_3_/CTF was significantly enhanced. This shows that the 0.25AgIO_3_/CTF photocatalyst generates more carriers under effective illumination conditions. The entire icon also reflects the characteristic quartet signal of the DMPO-OH and the characteristic peak signal of the DMPO-SO_4_^2−^ [11]. This indicates that -OH and SO_4_^2−^ are the main reactive oxygen species during the photocatalytic degradation process. In summary, the EPR test results prove that 0.25AgIO_3_/CTF will produce SO_4_^2−^, H^+^, and ·OH active substances during the degradation reaction and are related to the degradation process of pollutants.

**Figure 9:**
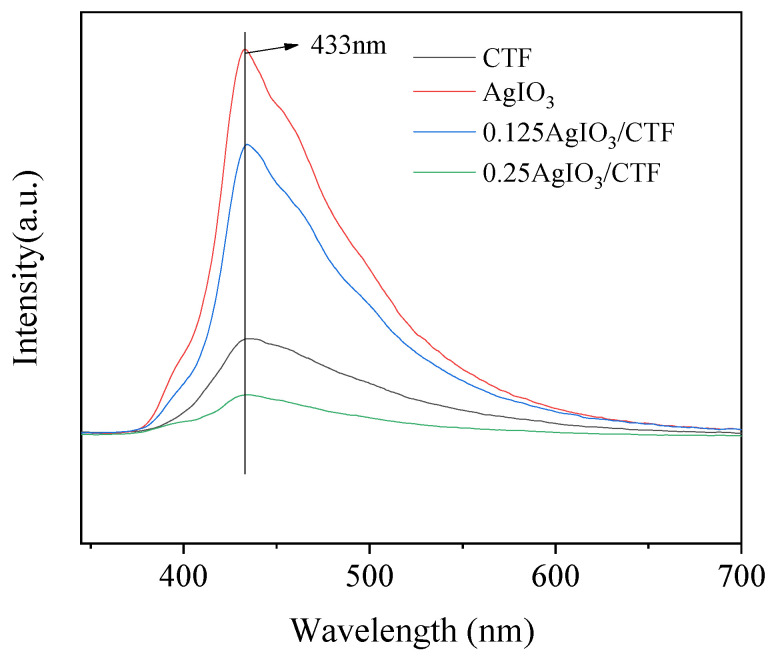
Stable photoluminescence spectra of CTF, AgIO_3_, and x-AgIO_3_/CTF.

Under light conditions, the steady-state photoluminescence spectra of the 0.25AgIO_3_/CTF and 0.125AgIO_3_/CTF; AgIO_3_; and CTF were further analyzed through fluorescence quenching and fluorescence lifetime experiments, as shown in Figure 9. At 433 nm, when the CTF is complexed with AgIO_3_, the AgIO_3_ has a strong emission peak at 433 nm. When the CTF forms a heterojunction with the AgIO_3_, photoinduced charges can be effectively separated. The order of luminescence intensity is AgIO_3_ > 0.125AgIO_3_/CTF > CTF > 0.25AgIO_3_/CTF, which is consistent with the above observed results of the photocatalytic activity. The combination of AgIO_3_ and CTF can reduce the emission peak, but the compound ratio of AgIO_3_ and CTF still needs to be explored [1,12,13].

**Figure 10:**
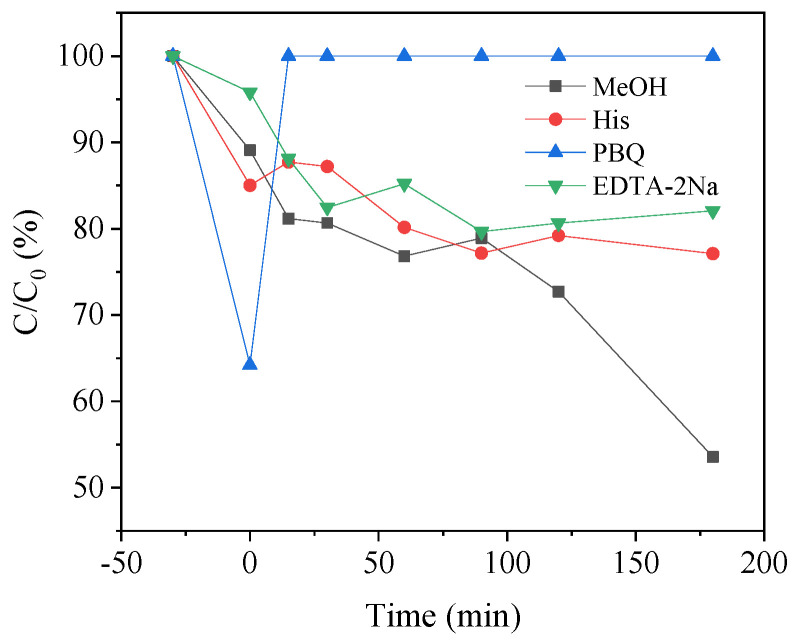
Inhibiting effects of quenching agents on carbamazepine degradation in 0.25AgIO_3_/CTF/vis/PMS system.

In order to further clarify the active substances involved in the photocatalytic process, free radical capture experiments were performed. In this experiment, disodium ethylenediaminetetraacetate (EDTA-2Na), methanol (MeOH), p-benzoquinone (PBQ), and histidine (His) were used as photogenerated holes (h^+^) and hydroxyl radicals (·OH). Furthermore, scavengers such as sulfate radicals (·SO_4_^2−^), superoxide radicals (·O^2−^), and singlet oxygen (1O_2_) were used. The experimental results are shown in Figure 10. During the degradation of carbamazepine by 0.25AgIO_3_/CTF, the degradation rate did not change significantly after adding the PBQ. This indicates that ·O^2−^ is not the main active species in the degradation of carbamazepine with this catalyst. After 180 min of illumination, the degradation rates of carbamazepine were 17.72% (EDTA-2Na), 22.80% (His), and 46.62% (MeOH), respectively. The results show that in the entire carbamazepine photocatalytic degradation system, ·OH and ·SO_4_^2−^ are the main active groups, and 1O_2_ and H^+^ play a smaller role.

**Figure 11:**
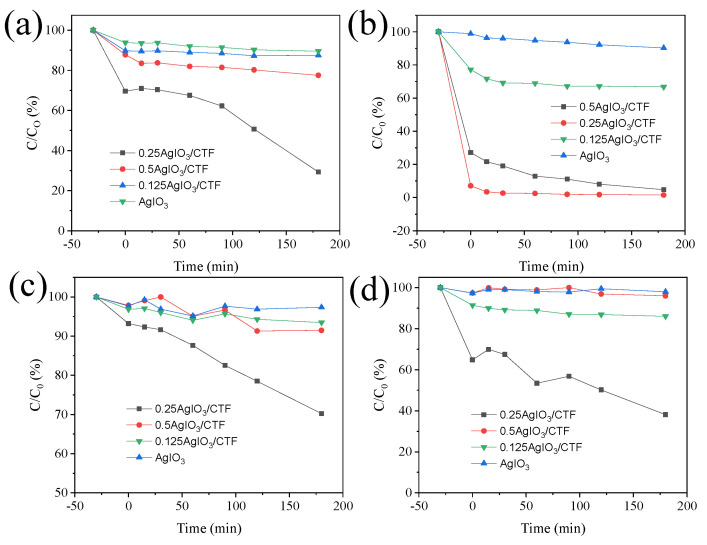
Degradation of different organic pollutants via photocatalysts/PMS/vis system: (**a**) phenol, (**b**) p-chlorophenol, (**c**) carbamazepine, and (**d**) paracetamol.

A degradation diagram of different composite catalysts for the simulated pollutants of paracetamol solution, carbamazepine solution, phenol solution, and p-chlorophenol solution is shown in Figure 11. It can be seen from the figure that these Ag element composite catalysts have varying degrees of degradation effects on the simulated polluted solution. As time goes by, the content of the contaminants in the solution gradually decreases. By calculating the degradation rates of the two catalysts for the two polluted solutions, the degradation rates of AgIO_3_ for the paracetamol solution, carbamazepine solution, phenol solution, and p-chlorophenol solution were 22.45%, 9.76%, 8.52%, and 3.96%, respectively. The degradation rates of 0.25AgIO_3_/CTF for the paracetamol solution, carbamazepine solution, phenol solution, and p-chlorophenol solution were 70.69%, 98.45%, 29.78%, and 61.85%, respectively. The photocatalytic degradation performance of the x-AgIO_3_/CTF composite material on the target contaminated solution was significantly higher than that of pure AgIO_3_. This shows that the lamellar structure of the CTF plays a good dispersion role in nano-spherical AgIO_3_ and increases the reactive sites. The formation of the heterostructure of the x-AgIO_3_/CTF composite material reduces the recombination rate of the photogenerated electrons and holes and greatly improves the photocatalytic activity. It has a good degradation effect on most pollutants under visible light irradiation and has a wide range of applications. In addition, the photocatalytic degradation of organic pollutants via different systems is demonstrated in Table 1; by comparison, the photocatalyst 0.25AgIO_3_/CTF prepared in this work has a higher degradation efficiency and a faster degradation rate for organic pollutants.

**Figure 12:**
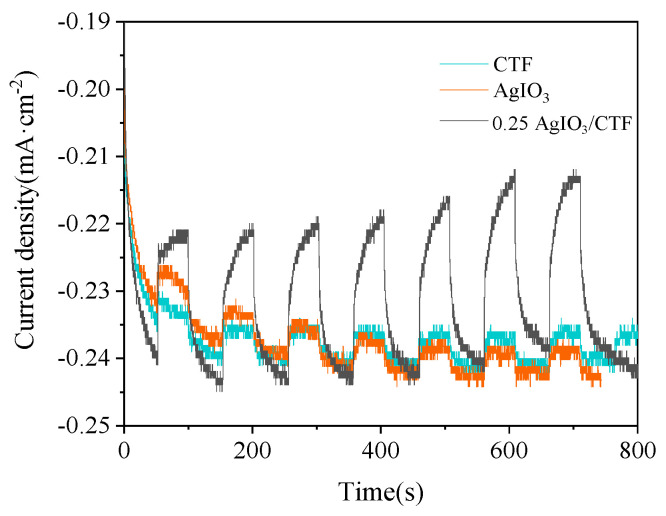
Periodic ON/OFF photocurrent response of CTF, AgIO_3_ and 0.25AgIO_3_/CTF.

In order to evaluate the transmission efficiency of photocarriers (photoexcited electrons and holes) and the generation of a photocurrent in the catalyst, photocurrent tests were conducted on the CTF, 0.25AgIO_3_/CTF, and AgIO_3_. The 0.25AgIO_3_/CTF was improved compared to the original CTF. Figure 12 shows the photocurrent test results of the CTF, 0.25AgIO_3_/CTF, and AgIO_3_. The results show that the photocurrent response intensity of the CTF monomer and AgIO_3_ monomer is smaller, which shows that the electron–hole separation efficiency of the two is relatively low. The photocurrent response intensity of the 0.25AgIO_3_/CTF composite material increased significantly. The 0.25AgIO_3_/CTF achieved a transient photocurrent and further enhanced the photocurrent. This finding confirms that the combination of CTF and AgIO_3_ can promote the generation and mobility of photoexcited charges under visible light irradiation. In addition, the arc radius of the 0.25AgIO_3_/CTF in the EIS image is much smaller than that of the CTF catalyst, indicating that the sample has extremely low resistance to the charge transfer in the framework, benefiting from the reduction of the photoinduced hole separation and the photocarrier recombination efficiency [13].

**Figure 13:**
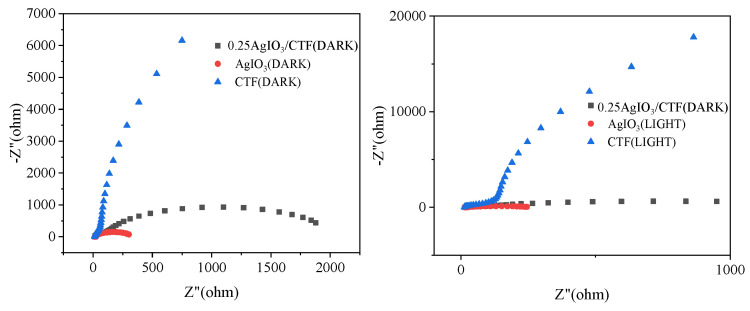
EIS Nyquist plots measured under the open-circle potential of CTF, AgIO_3_ and 0.25AgIO_3_/CTF.

To evaluate the kinetics of the charge transfer in this heterostructure composite, EIS tests were performed. The size of the arc radius on the Nyquist curve can reflect the photoreaction rate of the corresponding catalyst on the electrode surface. The smaller the arc diameter, the smaller the hindrance effect [14]. This shows that the smaller the charge transfer resistance Rct, the stronger the conductive performance of the electrode. A smaller arc radius indicates a lower interfacial layer resistance at the electrode surface. It can be seen from Figure 13 that the photocurrent of the composite photocatalyst film is significantly higher than that of the single catalyst film. This shows that the combination of x-AgIO_3_/CTF effectively inhibits the recombination of the photogenerated electron and hole pairs of a single catalyst, thereby increasing its photocurrent intensity. This also indicates that its ability to photocatalytically remove pollutants may also be improved. At the same time, the 0.25AgIO_3_/CTF shows the smallest arc radius compared to the AgIO_3_ and CTF, which indicates that the charge transfer resistance on the surface of the 0.25AgIO_3_/CTF is reduced. This, therefore, results in an efficient electron–hole pair separation, which is consistent with the i–t results. The above results confirm that the 0.25AgIO_3_/CTF photocatalyst has the highest separation efficiency and migration efficiency during the photocatalytic reaction process, thus giving it a better photocatalytic performance.

**Figure 14:**
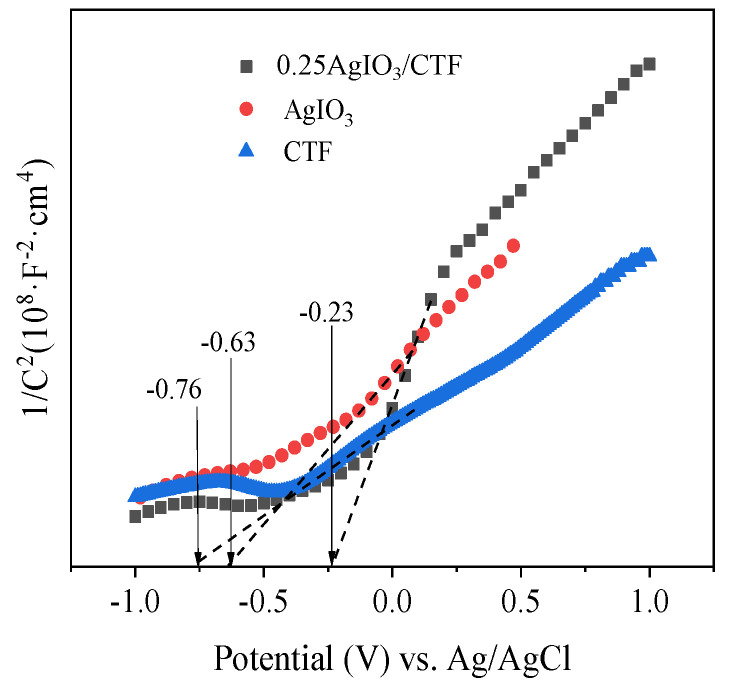
Mott–Schottky plots spectra of CTF, AgIO_3_, and 0.25AgIO_3_/CTF.

In order to better understand the mechanism of the CBZ-accelerated photocatalytic degradation in the 0.25AgIO_3_/CTF/vis/PMS system, the energy band structure of the 0.25AgIO_3_/CTF with respect to the photoinduced carrier redox ability was evaluated. Typical Mott–Schottky plots for various catalysts measured in the dark at a frequency of 100 Hz are shown in Figure 14. The positive slope of the 1/C^2^ values obtained indicates that each catalyst has n-type semiconductor properties. The flat band potentials of the CTF, AgIO_3_, and 0.25AgIO_3_/CTF samples were approximately −0.76, −0.63, and −0.23 V, respectively. Based on the relationship between the Ag/AgCl electrode and the normal hydrogen electrode (NHE), it can be calculated that the flat band potentials of each catalyst are −0.56, −0.43, and −0.03 V, respectively, compared to the normal hydrogen electrode (NHE). It has been reported that the flat band potential of n-type semiconductors is usually 0–0.1 V higher than the conduction band potential [15]. For organic semiconductor materials, the lowest unoccupied molecular orbital (LUMO) and the highest occupied molecular orbital (HOMO) are equivalent to the conduction band (CB) and the valence band (VB) of inorganic semiconductor materials, respectively [16]. Therefore, combined with the UV-visible diffuse reflectance spectrum of the sample, the conduction band and valence band potential of each sample relative to the standard hydrogen electrode potential can be obtained. Because the valence band potential energy of the 0.25AgIO_3_/CTF heterojunction photocatalyst is more negative than the redox potential energy of SO_4_^−^/SO_4_^2−^ (2.5–3.1 V vs. NHE) [17], the photogenerated electrons can be captured with PMS, thereby producing ·SO_4_^−^.

**Scheme 1:**
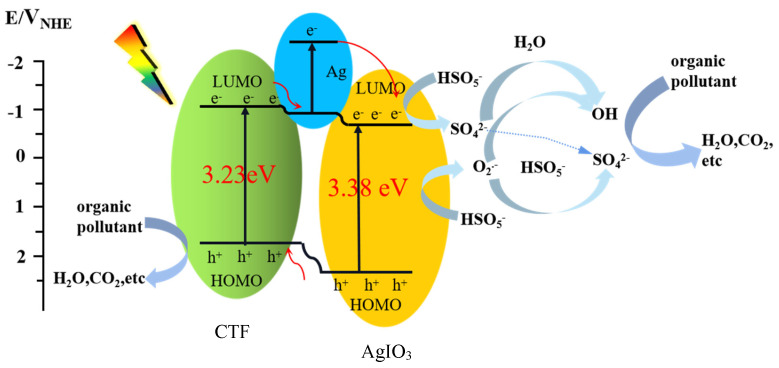
A schematic representation of the CTF/AgIO_3_ heterojunction photocatalyst adsorption–photocatalytic degradation of organic pollutants.

The characteristics of x-AgIO_3_/CTF meet the requirements for improving the photocatalytic performance. More specifically, the x-AgIO_3_/CTF increases the band edge to narrow the band gap and expands the visible light absorption boundary [18,19,20]. Due to the existence of elemental Ag, its plasmon resonance effect, and good conductivity, it can also generate photogenerated charges and quickly transfer electrons [18]. The existence of the heterojunction shortens the transmission distance of charges to the surface, thereby enhancing light capture and reducing the recombination probability of photoexcited carriers. As shown in Scheme 1, under the irradiation of the visible light, after electrons are excited, holes will be left in the valence band, but photogenerated electrons and holes are easy to recombine [21,22]. Due to the formation of a p–n heterojunction structure between the CTF and AgIO_3_, the valence band energy level of the AgIO_3_ is higher than that of the CTF. Holes will easily transfer to the CTF, thus inhibiting the recombination of the photogenerated electron–hole pairs inside the AgIO_3_ [23,24,25]. At the same time, due to the existence of elemental Ag, its plasmon resonance effect, and good conductivity, it can also generate photogenerated charges and quickly transfer electrons [26]. Taking advantage of the difference in the energy level positions between the CTF and AgIO_3_ and the presence of the elemental Ag, the recombination of the photogenerated electron–hole pairs inside the composite material is ultimately effectively suppressed and the photocatalytic activity of the composite material is improved. Under this condition, the holes (h^+^) react with HO^−^ in water to produce hydroxyl radicals (·OH) and the electrons react with dissolved oxygen (O_2_) in water to produce superoxide radicals (·O^2−^). After the introduction of the PMS, the separation of the carriers is further accelerated, due to the rapid capture of the photogenerated electrons. The PMS itself decomposes into various reactive substances through a series of chain reactions, and photooxidation (mainly h^+^) and PMS chemical activation (mainly ·OH and ·SO_4_^−^) are introduced to collaboratively remove organic pollutants. Because the PMS (1.82 V vs. NHE) is much greater than the oxidizing property of oxygen (1.23 V vs. NHE), the e^−^ on the conduction band is thermodynamically more likely to be captured by the PMS and then activated to generate free radicals (HSO_5_^−^ + e^−^→·SO_4_^−^ + OH^−^;·SO_4_^−^ + H_2_O→·OH + SO_4_^2−^ + H^+^). Since the redox potential of O_2_^−^ (0.91 V) is much lower than that of ·OH (1.9–2.7 V) and ·SO_4_^−^ (2.5–3.1 V), it may be less reactive with organic contaminants in aqueous solutions [27,28]. Therefore, using ·O_2_^−^ as an intermediate, other reaction species ·OH (HSO_5_^−^ +·O_2_−→·OH + SO_4_^2−^ + O_2_), SO_4_- (HSO_5_^−^ +·O_2_^−^→OH- + SO_4_^2−^ + O_2_), and 1O_2_ (2·O_2_^−^ + 2H_2_O→1O_2_ + H_2_O_2_ + 2OH^−^) are formed. These free radicals react with organic pollutants pre-adsorbed on the catalyst surface, causing the organic pollutants to be redox-degraded into inorganic small molecules (H_2_O, CO_2_, etc.) [29,30].

## 4. Conclusions

In this paper, a series of AgIO_3_/CTF catalysts with a heterojunction structure were successfully synthesized. The analysis of the composite photocatalyst using XRD, FTIR, and SEM shows that the reaction between AgIO_3_ powder and bulk CTF can successfully form a AgIO_3_/CTF composite photocatalyst material system, in which the crystallinity of the catalyst is good and a heterojunction structure is formed between the AgIO_3_ and the CTF. Compared with a single AgIO_3_, a AgIO_3_/CTF heterojunction photocatalyst significantly improves the degradation effect of organic pollutants. Among them, the degradation effect of 0.25AgIO_3_/CTF is the best. The reaction rate constants of phenol, p-chlorophenol, carbamazepine, and paracetamol are 0.00163, 0.00381, 0.0146, and 0.00482, respectively, which are 3.41, 25.07, 30.04, and 5.18 times that of the single catalyst AgIO_3_. The formation of a heterojunction between the AgIO_3_ and the CTF, driven by the built-in electric field, leads to the effective separation of the photogenerated electron–hole pairs and enhances the degradation efficiency of the organic pollutants. In addition, due to the LSPR effect, the Ag nanoparticles demonstrate significant absorption in the visible light region, playing a crucial role in the highly efficient photocatalytic reduction of antibiotics.

## Figures and Tables

**Table 1:** The catalytic performance comparison of recently reported photocatalysts.

Photocatalyst	Catalyst Loading (g L^−1^)	PMS (mM)	Pollutants	TargetsConc. (mM)	Removal Efficiency % (min)	Ref.
RGO-CoFe_2_O_4_	0.4	0.1	4-Chlorophenol	0.077	~100 (60)	[1]
Pt/WO_3_	0.5	1	4-Chlorophenol	0.1	~86 (100)	[2]
This work	0.1	0.65	4-Chlorophenol	0.1	~100 (180)	
Ni-ZnS/g-C_3_N_4_	1	0	Paracetamol	0.03	~86 (100)	[5]
ZnAlCe-MMO	0.5	1	Paracetamol	0.01	~99 (240)	[6]
This work	0.1	0.65	Paracetamol	0.1	~78 (180)	
BiOCl/Fe_3_O_4_	0.6	0	Carbamazepine	0.008	~75 (60)	[8]
Ag/AgCl/Bi_4_Ti_3_O_12_	1	0	Carbamazepine	0.021	~82 (120)	[9]
TiO_2_@SiO_2_@Fe_3_O_4_	1	0	Carbamazepine	0.008	~71 (540)	[10]
This work	0.4	1.3	Carbamazepine	0.021	~72 (180)	

## Data Availability

The data presented in this study are available on request from the corresponding author.
